# Differences in dynamic functional connectivity between naturalistic music listening and rest in preadolescents

**DOI:** 10.3389/fnhum.2025.1651074

**Published:** 2025-09-11

**Authors:** Ruijiao Dai, Petri Toiviainen, Fulvia Francesca Campo, Pauline Cantou, Maria Celeste Fasano, Boris Kleber, Peter Vuust, Elvira Brattico

**Affiliations:** ^1^Centre of Excellence in Music, Mind, Body and Brain, Department of Music, Art and Cultural Studies, University of Jyväskylä, Jyväskylä, Finland; ^2^Department of Translational Biomedicine and Neuroscience, University of Bari Aldo Moro, Bari, Italy; ^3^Laboratory of Behavioural Neurology and Imaging of Cognition, Department of Neuroscience, Campus Biotech, University of Geneva, Geneva, Switzerland; ^4^Department of Psychology and Behavioral Sciences, Aarhus University, Aarhus, Denmark; ^5^Center for Music in the Brain, Department of Clinical Medicine, Aarhus University and The Royal Academy of Music Aarhus/Aalborg, Aarhus, Denmark; ^6^Department of Education, Psychology, Communication, University of Bari, Bari, Italy

**Keywords:** naturalistic music listening, dynamic functional connectivity, functional magnetic resonance imaging, preadolescents, neuroplasticity

## Abstract

**Introduction:**

Engagement with music is a significant aspect of adolescents' lives and this interest blossoms during preadolescence. Compared to the extensive body of research focused on adult music brain function, relatively few studies have examined the neural connections involved in music listening among adolescents and preadolescents. This study aims to investigate the dynamic patterns of brain functional connectivity and the transition processes during naturalistic music listening in preadolescents, measuring the transition of brain states from one time point to the next.

**Methods:**

We employed functional magnetic resonance imaging (fMRI) to measure brain activity of 24 healthy preadolescents aged 8 to 12 years during both music listening and rest conditions. Subsequently, we applied a dynamic functional connectivity analysis, Leading Eigenvector Dynamics Analysis (LEiDA), to extract distinct brain states (phase locking patterns) and the corresponding transition processes.

**Results:**

Findings show that occipital brain regions are actively engaged during music listening, possibly linked to attention regulation, visual imagery, and emotional processing. Additionally, we observed a more frequent transition from the default mode network (DMN) state to an orbitofrontal limbic state during music listening, evidencing cognitive shifts that facilitate emotional and reward processing. In contrast, during rest, we obtained a switch to the sensorimotor auditory network, suggesting intrinsic fluctuations in multiple networks.

**Discussion:**

These findings deepen our understanding of subcortical and frontal brain connectivity in preadolescents during music listening, with implications for integrating music into educational practices to support learning and cognitive development.

## 1 Introduction

Music plays a pivotal role in the lives of children and adolescents, serving as a major source of entertainment and social interaction. During adolescence—an inevitable period marked by significant emotional, cognitive, and psychological development (McLaughlin et al., 2015; Uzun, 2022)—music becomes a powerful medium for self-development and peer bonding. This growing engagement with music typically sprouts in preadolescence (around ages 9–12) (Miranda, 2013). During this transitional period from childhood to adulthood, the brain continues to mature, particularly in regions such as the prefrontal cortex and the limbic system (Casey et al., 2019; Thomas, 2016; Yurgelun-Todd, 2007). The frontal lobe is involved in a large number of executive functions related to behavior and emotional control (Rosso et al., 2004; Stuss, 2011), while the limbic system —including structures like the nucleus accumbens—plays a central role in memory, emotions, and reward processing (Ernst et al., 2005; Kumar and Jha, 2024; Rolls, 2015).

During this period of profound development, music listening significantly influences both emotion regulation and functional connectivity (FC) in the brain. Research indicates that music, particularly slow-rhythm and minor-key compositions, can enhance mental conditioning and alleviate tension in adolescents (Wang et al., 2018). Furthermore, a neuroimaging study reported enhancements in cerebral neural development and brain plasticity following pop music training in young individuals (Yu, 2018). In a recent neuroimaging study, Fasano et al. (2023) examined dynamic functional connectivity during music listening in preadolescents and found that, compared to silence, music listening significantly increased the occurrence of a functional network involving the medial orbitofrontal cortex and the ventromedial prefrontal cortex—regions critically involved in reward processing. Notably, preadolescents with greater sensitivity to musical reward exhibited more frequent transitions from a network involving the insula to this reward-related network, suggesting a coupling between interoceptive processing and hedonic evaluation.

Research on music listening has extensively documented its effects on adults, uncovering a range of cognitive, emotional, and social benefits, such as elicitation of emotions, regulation of arousal and facilitation of inter-person motor coordination (Blood et al., 1999; Koelsch, 2014; Mammarella et al., 2007; Patston and Tippett, 2011; Peretz and Zatorre, 2005; Zhang, 2020). Findings from human connectome studies suggest that the origins of these effects lie in changes of neural connectivity patterns in the brain rather than in the isolated activation of individual brain regions (Alluri et al., 2017; Burunat et al., 2015; Reybrouck et al., 2018; Toiviainen et al., 2014). For instance, functional magnetic resonance imaging (fMRI) studies have revealed synchronized brain responses through auditory, motor, frontal, and parietal cortex during music listening (Abrams et al., 2013), while electroencephalography studies have demonstrated functional connectivity between temporal, occipital, and prefrontal regions (Zhu et al., 2020). A review by Reybrouck et al. (2018) summarized the functional connectivity patterns related to music listening and identified the key players: neural networks connecting the auditory cortex with the reward system, and or brain circuits related to mind-wandering, namely the default mode network (DMN), as well as frontoparietal and limbic networks.

Although neuroimaging research has significantly advanced our understanding of FC between brain regions during music listening, research focusing on preadolescents remains limited. Given the substantial neurodevelopmental changes occurring during this period, further investigation into how music modulates brain networks in younger populations is crucial in order to elucidate the central role of music listening in this age period (Miranda, 2013; Sachs et al., 2016; Tierney et al., 2015).

In the present study, we investigated brain connectivity changes during music listening in preadolescents by using a novel dynamic FC (dFC) approach known as Leading Eigenvector Dynamics Analysis (LEiDA). Unlike traditional static FC methods, the dFC captures temporal variability in brain connectivity, allowing for the identification of transient network states and their dynamic transitions over time. LEiDA offers advantages over traditional dFC correlation methods, including dimensionality reduction, high-frequency noise robustness, and improved temporal resolution (Cabral et al., 2017; Lord et al., 2019). Recent studies have yielded valuable insights through the application of the LEiDA methodology in the context of naturalistic music listening. In particular, Dai et al. (2024) employed this method to investigate the neural dynamics underlying aesthetic experiences during naturalistic music listening in adults, identifying functional connectivity patterns and their temporal evolution during music listening (Dai et al., 2024). Similarly, Fasano et al. (2023) utilized LEiDA to examine dynamic brain connectivity in preadolescents during music listening, revealing the recruitment of a network involving brain regions associated with hedonic and emotional processing. In that study, two short medleys of violin themes were composed specifically for children as the musical stimuli. The pieces were selected for their simplicity, unfamiliarity, and ease of learning, as the study formed part of a longitudinal investigation into the neural effects of music training; however, the comparison condition of rest was very short compared with the music listening condition, representing a limitation in the methodological design. In the present study, we aimed to extend those findings by using a musically richer and more emotionally expressive and varying piece, Adiòs Nonino, which we anticipated would enhance the engagement in young listeners. Additionally, we introduced a comparably long rest condition to thoroughly examine dynamic functional changes between music listening and baseline. Existing research has demonstrated the potential of LEiDA to capture temporally evolving brain states linked to emotional and hedonic aspects of music perception. Building on this prior work, by using LEiDA, our study aims to identify specific FC patterns and dynamic interaction processes during naturalistic music listening in the preadolescent brain.

## 2 Materials and methods

### 2.1 Participants

A total of 33 healthy Danish preadolescents (aged 8–12 years) were recruited in the experiment. Inclusion criteria required that participants had no metal in the body, no hearing, neurological, or psychological disorders, no chronic medication use, no tattoos or recent permanent coloring no claustrophobia. All experimental participants and their guardians provided informed consent before the experiment. This study was part of a longitudinal investigation on music training involving children who were starting a music school and those who were on the waiting list. Some of them were recruited from an advertisement on Facebook. All participants were studying in 6 of the local public elementary schools in Aarhus. The study was approved by the Danish Research Ethics Committees (Scientific Ethics Committees for the Central Denmark Region).

Nine participants were excluded due to: (1) an unfinished experiment; (2) massive motion artifacts; or (3) incomplete datasets. Consequently, 24 preadolescents, without any prior music training background, were included in this study (mean age: 10.63 ± 1.29 SD; 11 males).

### 2.2 Stimuli

During fMRI scanning, participants completed a music-listening task in which they were instructed to listen to the symphonic part of the piece *Adiòs Nonino*, an instrumental *tango nuevo* composed by the Argentinian composer Astor Piazzolla in 1959 (hereafter referred to as “Piazzolla”). This piece is known from previous studies to evoke a strong emotional response and robust neural engagement, as demonstrated by behavioral and neuroimaging studies employing fMRI and magnetoencephalography (MEG) (Burunat et al., 2014; Alluri et al., 2017; Zhu et al., 2020). While we were aware of the complexity of the Piazzolla piece, we selected it due to its strong emotional connotations, which we anticipated would enhance engagement even in young listeners. Musical stimuli were presented using an MRI-compatible music-playing system (OptoACTIVE II, Optoacoustic Ltd. Mazor, Israel).

### 2.3 fMRI data acquisition

The fMRI data were acquired at the MindLab facilities of the Danish Neuroscience Center in Aarhus, Denmark. The total scanning session lasted 43 min and comprised four distinct sequences: resting-state fMRI (8 min), a cognitive task (12 min), a music-listening task (4 min), and an anatomical MP2RAGE sequence (11 min). The results concerning the cognitive task are reported in a separate manuscript (Cantou et al., submitted). The fMRI study was conducted using a standard 32-channel head-neck coil with a 3T Siemens Prisma fit scanner at the Center for Music in the Brain (MIB), Department of Clinical Medicine, Aarhus University. Foam pads were placed around participants' heads in order to prevent head movement artifacts and dampen scanner noise. A gradient echo planar imaging (EPI) sequence (TE = 29.6 ms; TR = 1s; voxel size = 2.5 mm^3^), including 8 min of rest and 4 min of music listening, was acquired. During the rest period, participants were instructed to keep their eyes open and remain awake. During the music listening period, participants listened to the music piece with the volume individually adjusted to a comfortably audible level before the session. Additionally, high-resolution 3D T1-weighted structural images (192 slices; TE = 3.47 ms; TR = 6.5s; voxel size = 0.9 mm^3^) were obtained for each participant using an MP2RAGE sequence, during which short cartoons ‘The Lego Story' were played for every participant to help them stay focused and minimize movement artifacts.

### 2.4 Preprocessing

The fMRI data obtained during 4 min of listening and 8 min of rest were preprocessed using SPM12 (Multivariate Exploratory Linear Optimized Decomposition into Independent Components) version 3.15, based on MATLAB R2018b (MathWorks). Firstly, the first 10 time points were removed to prevent possible signal instability at the beginning of the signal acquisition process. Then, slice timing and motion correction were performed to align the acquisition times of different brain slices and mitigate any head movements during the scan. Segmentation was then conducted to identify and separate different tissue types into gray matter, white matter, and cerebrospinal fluid. Normalization of individual functional EPI to an average brain template in Montreal Neurological Institute (MNI) space for children aged 7.5 to 13.5 years was performed, based on Fonov et al. (2011)—details can be found at https://www.bic.mni.mcgill.ca/ServicesAtlases/NIHPD-obj1. Spatial smoothing was applied using a 6 mm full-width at half-maximum (FWHM) Gaussian kernel. Additionally, a band-pass filter was utilized between 0.01 and 0.1 Hz, the typical resting state filtering frequency range, to isolate low-frequency fluctuations while effectively removing physiological components, as described by Chen and Glover (2015). Finally, the time course of the blood oxygenation level-dependent (BOLD) signal was extracted into *N* = 90 brain regions according to the Automated Anatomical Labeling (AAL) method.

### 2.5 Leading Eigenvector Dynamics Analysis

In this study, we apply the LEiDA algorithm to capture the dominant FC pattern of BOLD signals. A time-resolved dFC matrix was obtained by calculating the phase coherence of the BOLD signals using the Hilbert transform. The phase coherence of the BOLD signals *x*(*t*) can be expressed as *x*(*t*) = *a*(*t*)cos[φ(*t*)], where *a*(*t*) represents the instantaneous envelope and φ(*t*) represents the instantaneous phase. Subsequently, the dFC matrix, *dFC*(*n, p, t*), which calculates the phase coherence between brain regions *n* and *p* at time *t* can be expressed as the following equation:


(1) $$\begin{array}{l} dFC\left(n,p,t\right)=\cos\left[\varphi \left(n,t\right)-\varphi \left(p,t\right)\right] \end{array}$$

where *n, p* = 1, 2, …, *N*, *N* represents the number of brain regions (*N* = 90) and *t* = 1, 2, …, *T*, *T* represents the length of the time courses of the BOLD signal. When the two brain regions, *n* and *p*, have an aligned phase at time *t*, the dFC value is: *dFC*(*n, p, t*) = 1. Conversely, if the two brain regions have an anti-aligned phase at time *t*, the dFC value is: *dFC*(*n, p, t*) = −1. If the two brain regions are orthogonal, the dFC value is: *dFC*(*n, p, t*) = 0.

The dFC matrix is an *N*×*N* symmetric matrix due to the nondirectional nature of phase coherence at each time point. Consequently, the upper or lower triangular portion of the matrix contains all the essential characteristics of the dFC states.

To reduce the dimensionality from *N*(*N*−1)/2 to *N*, the leading eigenvector *V*_1_(*t*) is extracted, capturing the predominant FC patterns of each dFC matrix. The dominant FC matrix can be reconstructed using the outer product $V_{1}V_{1}^{T}$ (*N*×*N*). Each *V*_1_ contains *N* elements, where each element represents a brain region.

The sign of each element in V_1_ indicates community structure: when all the elements share the same sign, all brain belongs to a community; otherwise, if elements have both positive or negative signs, the phases of the BOLD signal are divided into two distinct communities (Newman, 2006; Cabral et al., 2017; Figueroa et al., 2019). Due to this property, *V* and −*V* exhibit the same relative brain connectivity relationship. To ensure consistency in this study, we standardized each leading eigenvector such that most of its elements were negative, multiplying by −1 when necessary. The magnitude of each element reflects the “strength” of the corresponding brain regions within its community (Newman, 2006).

### 2.6 Phase locking states

To examine dynamic functional connectivity during music listening in preadolescents, we used *k*-means clustering to identify specific phase-locking (PL) states that differed significantly between music listening and rest. To this end, all leading eigenvectors were collected across all time points and participants, totaling 17,352 leading eigenvectors (490 time points for the rest and 233 time points for the music listening condition across 24 participants). The *k*-means clustering algorithm categorized all leading eigenvectors into *k* clusters, with each cluster representing a distinct PL state. To mitigate false positives due to excessively large *k* values, clustering was performed with *k* values ranging from 2 to 10. The corresponding PL states were obtained for each *k* value. To investigate the dynamic brain connectivity patterns between music listening and rest in preadolescents, dFC patterns of both states were statistically compared using a *t*-test. To assess the statistical significance of each *t*-test comparison, we performed 10,000 permutation tests, which involved randomly shuffling group labels to create a null distribution and determine whether observed differences were stronger than chance. This non-parametric approach avoids distributional assumptions while strictly controlling for false positives.

## 3 Results

### 3.1 Detection of the recurrent PL states

**Figure 1:**
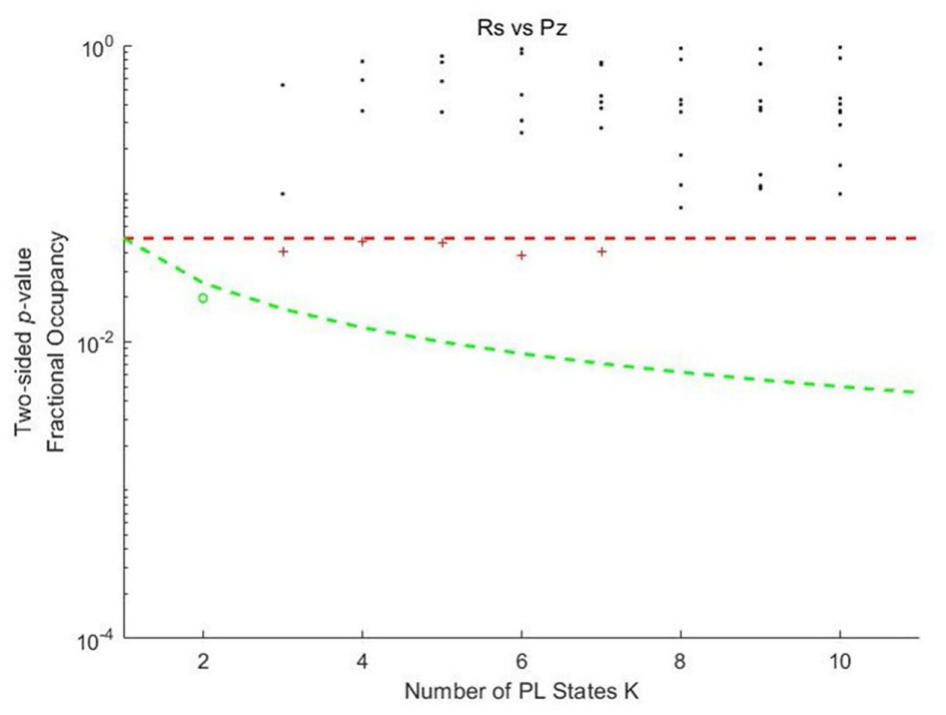
Significance values for the probabilities of Phase locking states in each *k*-means clustering model as a function of *k* between music listening and rest. Pz, Piazzolla (representing the music listening condition); Rs, rest condition. Each dot represents a *p*-value obtained from the permutation *t*-test, which assesses the probability of occurrence of each PL state between music listening and rest as a function of *k* in preadolescents. The red dashed line represents the uncorrected threshold (*p* < 0.05). The green dashed line represents the corrected threshold adjusted for the number of clusters (*p* < 0.05/*k*). Dot colors indicate significance levels. The black asterisks represent PL states with no significant differences in probability of occurrence. Red plus signs represent states that passed the uncorrected threshold but did not meet the corrected threshold. The green circles represent the states that passed the corrected threshold.

The probability of occurrences for each PL state during the music listening and rest conditions was determined using *k*-means clustering with *k* values ranging from 2 to 10. As shown in Figure 1, for *k* = 3, 4, 5, 6, and 7, one PL state exhibited a significant difference (< 0.05) between the music listening and rest conditions. When *k* = 2, the PL state showed the most significant difference (< 0.05/*k*) after correction for multiple comparisons, with *p*-values passing the threshold adjusted for the number of clusters.

### 3.2 PL states with significant differences

**Figure 2:**
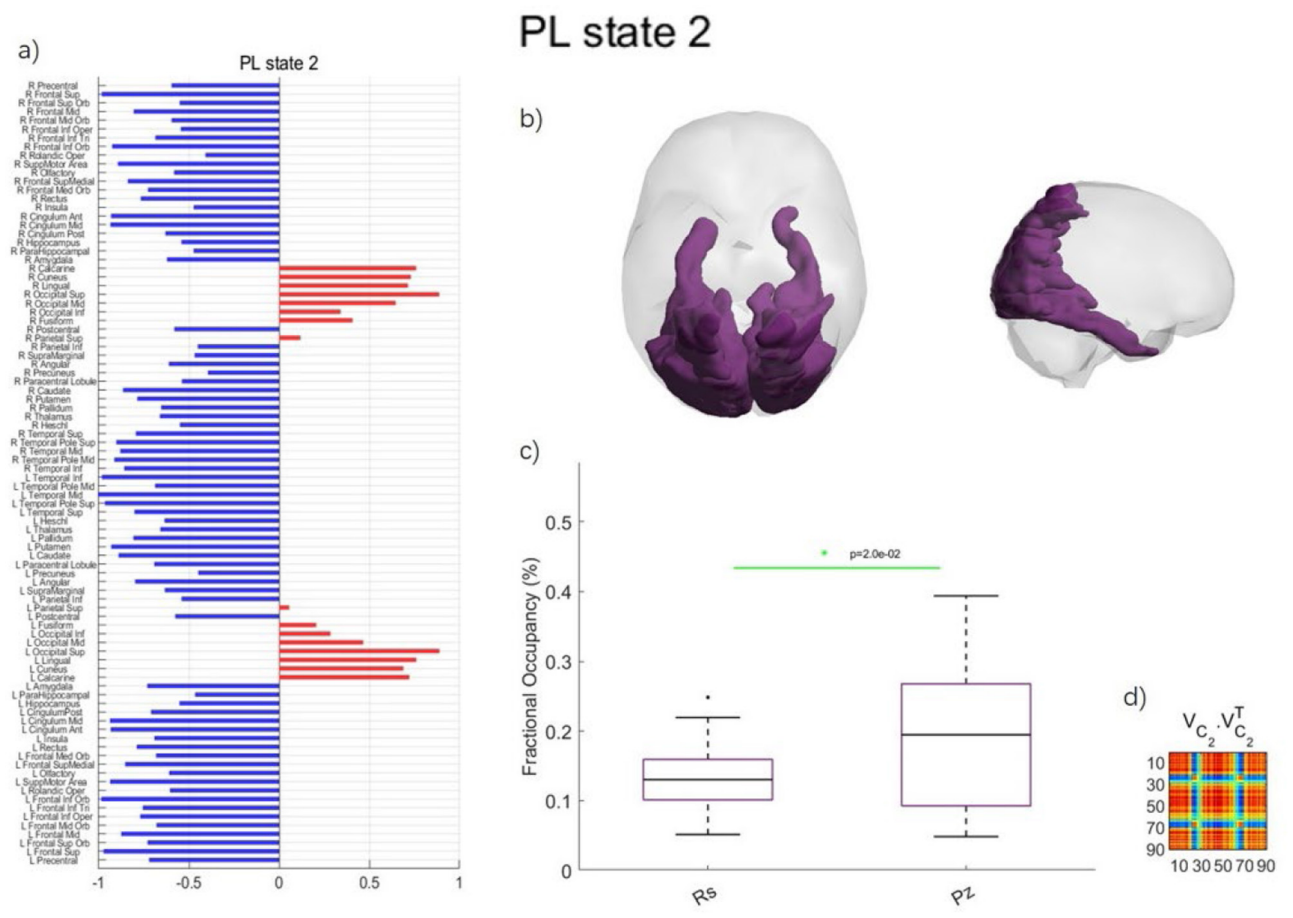
Phase locking state for *k* = 2 with the most significant differences between music listening and rest in preadolescents. **(a)** PL state represented in vector format. The signs of elements (blue or red) indicate brain regions belonging to two distinct communities. Regions with the same color form a connected community, and the magnitude of the elements indicates the “strength” of the corresponding brain regions within the community. R and L indicate the right or left hemisphere, respectively. **(b)** Axial and sagittal view of the PL state for *k* = 2. **(c)** Error bar charts displaying the probability of occurrence for each PL state between music listening (Pz, short for Piazzolla) and rest (Rs). **(d)** Outer product reconstruction of the dominant FC matrix calculated by $V_{1}V_{1}^{T}$.

In Figure 2, the PL state 2 for *k* = 2 exhibited the most significant differences in the probability of occurrence between music listening and rest.

As shown in Figure 2, PL state 2 includes the calcarine fissure and surrounding cortex, cuneus, lingual gyrus, occipital gyrus, fusiform gyrus, and superior parietal gyrus (SPG). The probability of occurrences of the PL state 2 was significantly different between music listening (18.73 ± 10.92%) and rest (16.78 ± 6.53%) in preadolescents (*p* = 0.0199).

**Figure 3:**
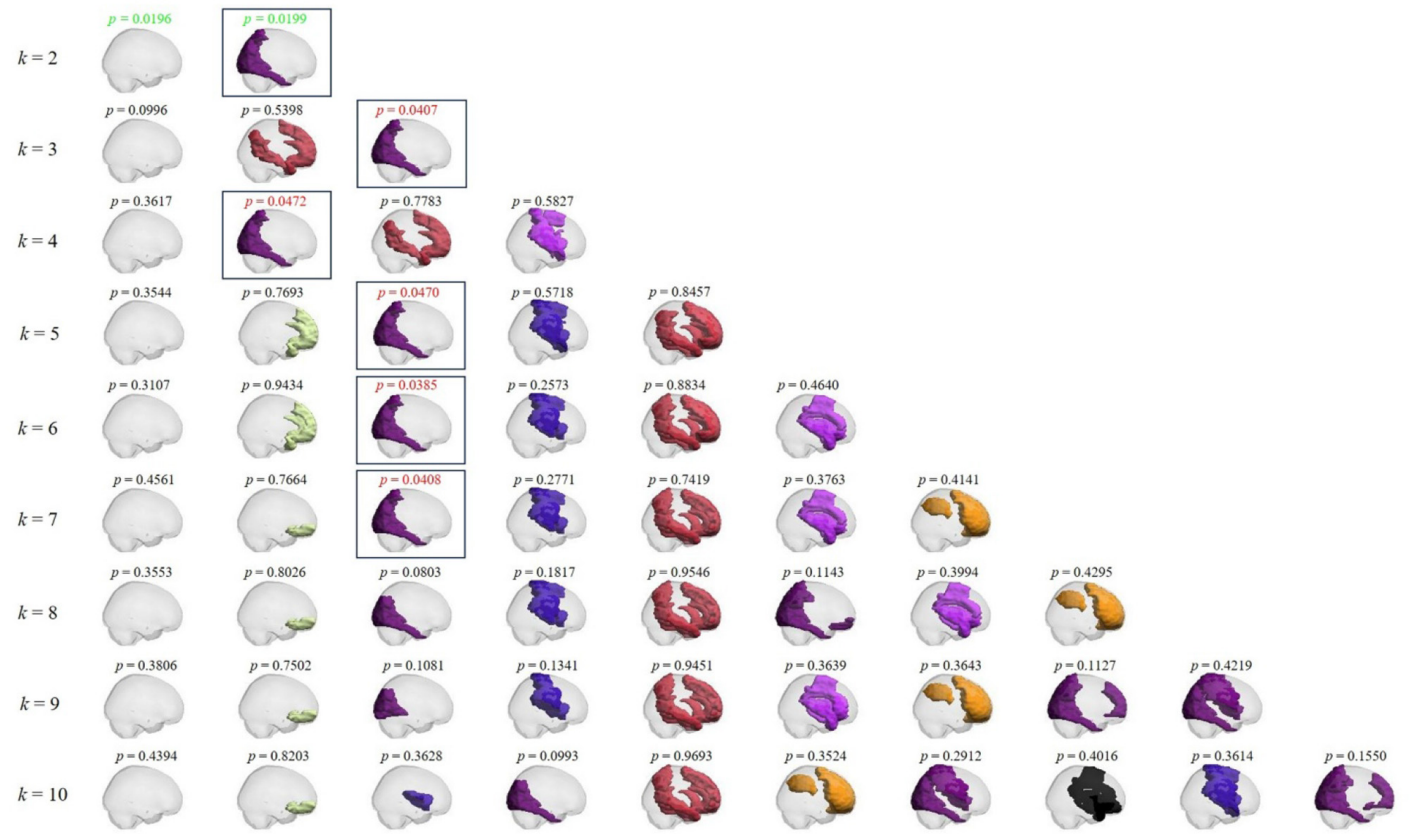
Phase locking states and the corresponding *p*-values between music listening and rest conditions in preadolescents as a function of *k*. From top to bottom: PL states obtained for each *k* value from 2 to 10 in sagittal view. Above each PL state, the corresponding *p*-value. Red *p-*values: *p* < 0.05; green *p*-values: *p* < 0.05/*k*. Boxes emphasize all PL states with significant differences across conditions.

Figure 3 presents all PL states obtained through *k*-means clustering from *k* = 2 to *k* = 10 between music listening and rest. The corresponding *p*-values are labeled above each PL state. Notably, all significantly different states (within square boxes) are identical across conditions, corresponding to the visual parieto-occipital brain network of PL state 2 for *k* = 2, demonstrating the robustness of the results.

In sum, the results illustrated in Figure 3 confirmed that all statistically significant PL states exhibited consistent properties at this clustering level.

### 3.3 Transition processes

**Figure 4:**
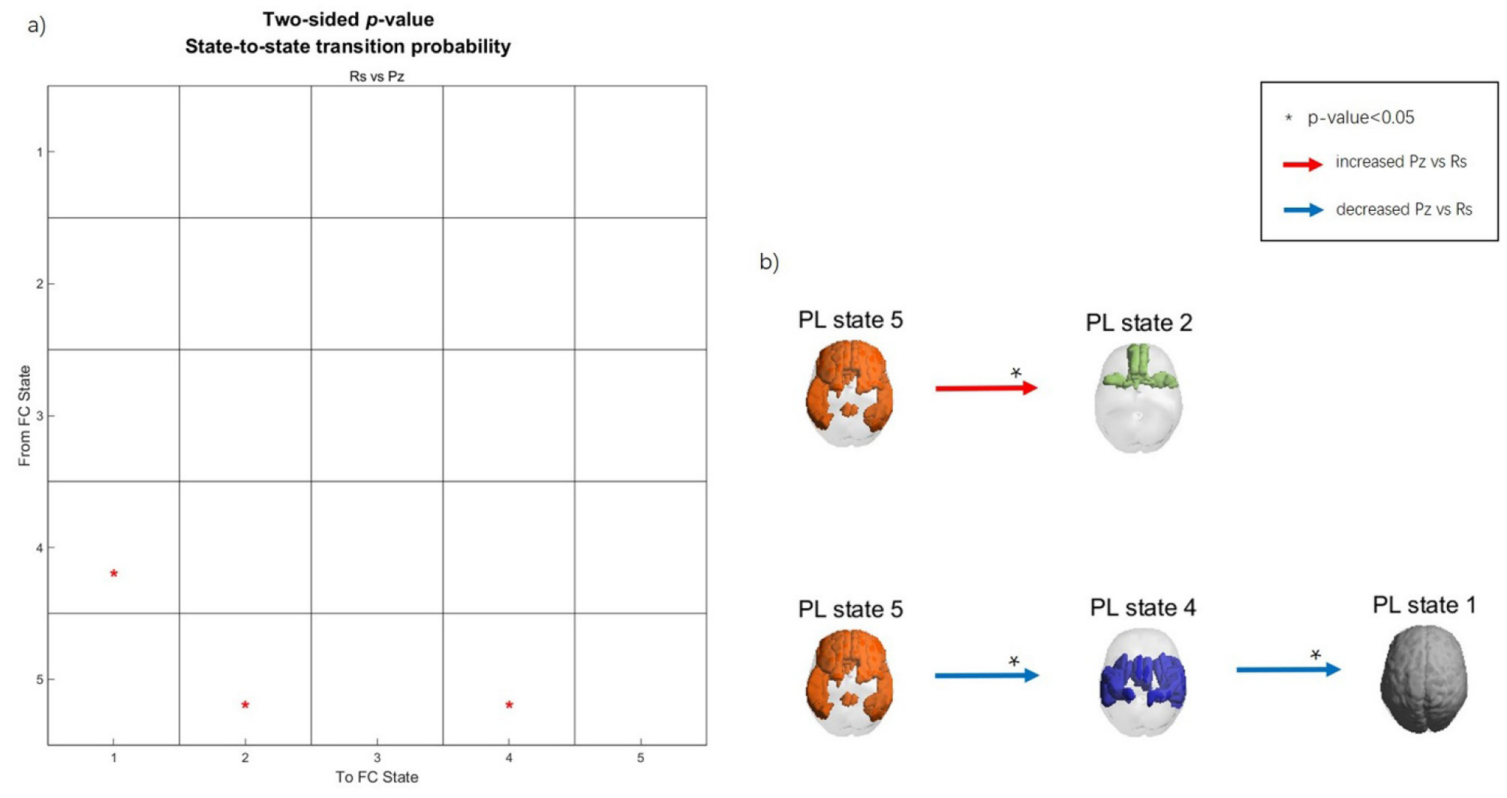
Transition process during music listening and rest in preadolescents. **(a)** Transition probability matrix. The Y-axis represents the previous time point, while the X-axis represents the next time point. **(b)** Spatial maps of the PL states and transition processes. Red arrows indicate increased transition probability during music listening compared to rest, whereas the blue arrows indicate decreased transition probability during music listening compared to rest. Asterisks (*) indicate significant differences between conditions (*p* < 0.05).

A PL state change from one time point to the next is considered a *transition*. We calculated the transition probability separately for the music listening and rest conditions in preadolescents. Subsequently, we compared the transition probabilities between these two states using a *t*-test (10,000 permutations). The transition probability matrix and the transition process are presented in Figure 4. Since our clustering findings obtained with *k* = 2 contained only two PL states, which would result in an oversimplified transition process, to properly identify the transition dynamics, we selected *k* = 5 clustering solution for subsequent analysis of transition probabilities.

As visible from Figure 3, PL state 1 represents the global state. PL state 2 includes the middle frontal gyrus (MFG), orbitofrontal cortex (OFC), gyrus rectus, olfactory cortex, middle temporal gyrus (MTG), and the left anterior cingulate cortex (ACC). PL state 4 includes the Rolandic operculum, supplementary motor area (SMA), insula, postcentral gyrus, supramarginal gyrus, putamen, Heschl gyrus, superior temporal gyrus (STG), and the left amygdala. PL state 5 includes the superior frontal gyrus (SFG), MFG, inferior frontal gyrus (IFG), gyrus rectus, ACC, posterior cingulate cortex (PCC), right inferior parietal gyrus (IPG), angular gyrus (AG), MTG, and the left inferior temporal gyrus.

During music listening, the transition probability from the frontoparietal PL state 5 to the orbitofrontal limbic PL state 2 increased significantly compared to rest. In contrast, during rest, there was a higher probability than during music listening for the transition from the frontoparietal PL state 5 to the sensory-motor PL state 4, followed by global state 1.

## 4 Discussion

In this study, we investigated the dynamic connectome in preadolescents during naturalistic music listening as compared with rest. Naturalistic music listening is a research method that studies how people engage with music in their everyday lives, aiming for high ecological validity. This is fundamentally different from naturalistic music, which is a genre of music created from sounds sourced from the natural environment. The first term describes a scientific approach to studying listening behavior, while the second describes an artistic approach to creating music. Our result revealed that one phase locking state occurred with higher probability during music listening as contrasted with rest across multiple clustering results. This PL state, including the calcarine fissure and surrounding cortex, cuneus, lingual gyrus, occipital gyrus, fusiform gyrus, and SPG, is typically associated with visual processing, high-level vision computations, spatial orientation, voluntary attention shifts, episodic memory retrieval, and high-level cognitive functions (Belliveau et al., 1991; Collignon et al., 2011; Sack, 2009; Zhang and Chiang-shan, 2012).

Our results indicated that brain regions involved in vision processing are also engaged during listening to naturalistic music in preadolescents. Such finding resonates with the mechanism of visual imagery in music-emotion induction, as described by Juslin and Västfjäll (2008)), which refers to the evocation of vivid mental pictures during music listening. According to their framework, when individuals engage with music, they may conjure vivid mental pictures that are associated with the themes, narratives, or even personal memories linked to the piece being heard, which in turn influences the listener's emotional state. An alternative mechanism that might cause early visual cortex recruitment during music listening is attention. Indeed, research shows that even in the absence of visual input, the mere attending to sounds can modulate activity in the visual cortex. This is supposed to be related to cross modal facilitation and attentional preparation in expectation of the sensory input to come, where the brain enhances sensory processing across modalities to support behavior (Cate et al., 2009; Lange et al., 2003). Moreover, the recruitment of visual cortices during auditory attention might derive from top-down modulation via frontoparietal networks for allowing sensory prioritization based on task relevance rather than modality (Macaluso and Driver, 2005).

Neuroimaging evidence supports the recruitment of visual regions during sound processing. A positron emission tomography (PET) study in adults (Satoh et al., 2015) investigated cognitive processing related to the perception of sound richness and revealed that the lateral occipital complex and fusiform gyrus are engaged in both visual and auditory processing. More specifically, fusiform gyrus activation increased with sound richness, while the lateral occipital complex participated in discriminating between melody and accompaniment. While these two regions are traditionally associated with visual processing, Satoh et al. demonstrated their involvement in auditory processing as well. Similarly, Liu et al. (2017) in a functional connectivity study also reported that the occipital gyrus, cuneus, and lingual gyrus were connected with each other during attentive music listening, which led to aesthetic evaluations. Additionally, Bravo et al. (2024) revealed that dissonant music enhances activity in the early visual cortex, indicating strong auditory-visual interactions.

Our findings are in line with the existing literature in highlighting that occipital brain regions might be more frequently connected during music listening than during rest, possibly reflecting their role in attention regulation and visual imagery associated with affective music processing. Remarkably, we obtained these findings with preadolescent children, further supporting the involvement of non-auditory processes during music listening even in developmental stages.

Furthermore, when considering the transition process, we observed more frequent transitions from the frontal lobe, temporal lobe, IPG, ACC, PCC, and AG to the MFG, OFC, MTG, and ACC during music listening compared to rest. These transitions suggest that, during music listening, there occur frequent switches from the DMN to the orbitofrontal limbic network. The frontal lobe, which includes the SFG, MFG, and IFG, contributes to higher-order cognitive processes, such as attention, working memory, planning, decision-making, cognitive control, language processing, and verbal memory during music listening (Tabei, 2015; Chan and Han, 2022; Tillmann et al., 2006). Furthermore, the OFC and ACC, which are components of the dopaminergic mesolimbic reward pathway, are involved in music-evoked pleasure (Koelsch, 2014; Zatorre and Salimpoor, 2013). These regions may facilitate sustained attention to music, analysis of musical structure, processing of complex musical patterns, reflection on the emotional experience elicited by the music, and integration of various musical elements.

The OFC plays a crucial role in music listening for both adults and preadolescents, acting as a central hub for emotional processing, reward evaluation, and social engagement (Blood and Zatorre, 2001; Menon and Levitin, 2005; Salimpoor and Zatorre, 2013). In adults, (Blood and Zatorre 2001) reported a network of brain regions, including OFC, ACC, insula, and ventral striatum during music appreciation. A recent fMRI study (Ma et al., 2024), using diffusion tensor imaging (DTI), revealed an increased passing probability of OFC in composers compared to the control group, suggesting that composers exhibit enhanced connectivity and integration, and a more efficient information transfer. The OFC is reported to be engaged in music listening, particularly in the context of aesthetic appreciation (Dai et al., 2024; Brattico et al., 2025; for a review, see Reybrouck et al., 2018). In preadolescents, Fasano et al. (2023), also using LEiDA as here, reported more frequent connectivity and switching of the OFC and ventromedial prefrontal cortex during music listening. In both demographic groups, OFC acts as a central hub of the reward system, playing a vital role in emotional processing, reward evaluation, memory, learning, and social engagement.

During rest, in contrast to music listening, we found the preadolescents' brain networks to frequently switch from DMN to the sensorimotor auditory network before reaching the global state. This finding extends what was obtained by Fasano et al. (2023) who did not include a lengthy resting condition, hence, could not investigate dynamic resting state connectivity in preadolescence. The DMN is particularly characterized by heightened activation and connection during wakeful rest, when individuals are not focused on external tasks (Greicius et al., 2003; Raichle and Snyder, 2007). Research using resting state fMRI has demonstrated the concurrent engagement of multiple networks, including those responsible for motor, sensory, visual, and language processing (Cordes et al., 2000; Fransson, 2005; Seeley et al., 2007). Additionally, our findings indicate the involvement of motor regions in preadolescents, which may reflect motor imagery, as preadolescents might struggle to remain still in the scanner during rest.

Analyses of the transition process further revealed that the salience network, auditory network, and sensorimotor network are simultaneously synchronized during rest. More specifically, the salience network engages the ACC, insula, amygdala, and prefrontal cortex; the auditory network mainly engages the STG and Heschl's gyrus; while the sensorimotor networks engage the parietal cortex, supplementary motor area, and basal ganglia. Previous studies have suggested that the brain networks related to motor, auditory, visual, and language processing exhibit intrinsic fluctuations during rest (Biswal et al., 1995; Cordes et al., 2000; Lowe et al., 1998).

One of the limitations of this study is the relatively small sample size. Researching adolescents can be particularly challenging due to various factors, including their developmental stage, varying levels of engagement and willingness to participate in studies, and the impact of external influences such as peer dynamics and family situations. These factors can lead to difficulties in recruiting a sufficiently diverse and representative sample. Furthermore, while this study explored the dynamics of brain networks during listening to an acoustically and musically complex piece, an Argentine tango, we nevertheless focused on just one piece and one specific style of music. Future studies on the musical experience in preadolescence and adolescence should include a much larger sample of participants and a wider variety of musical styles, including popular songs, which may be more suitable for the developmental stage of the participants. Additionally, future studies will also focus on different age groups to enable more direct comparisons of how listeners of different ages respond to music.

## 5 Conclusion

In this study, we employed LEiDA to investigate functional connectivity patterns and dynamic interaction processes in preadolescents during naturalistic music listening and rest. Our findings suggest that visual areas are actively engaged during music listening in preadolescents. This engagement may reflect their role in regulating attention and generating visual imagery, which are associated with the emotional processing of music. These observations support the notion that music listening involves a wide range of non-auditory processes, even in the developmental stage of preadolescence. Additionally, this study provides additional evidence for music's ability to connect higher-order cognitive brain regions with reward-related orbitofrontal areas in preadolescence. Taken together, these findings suggest that the informed use of music can be a powerful tool. For instance, selecting pieces that are emotionally engaging to activate reward pathways could enhance motivation and reinforce learning. Moreover, as music listening is a dynamic cognitive task that exercises brain flexibility, it is not merely an auditory art form but a full-brain workout. When used strategically, it can foster motivation, emotional regulation, and core cognitive skills in developing preadolescents. Overall, these findings enhance our understanding of brain activity in preadolescents during music listening and may inform the integration of music into educational practices for youth, emphasizing the informed use of music as a tool for learning and cognitive development.

## Data Availability

The raw data supporting the conclusions of this article will be made available by the authors, without undue reservation.
